# Au-NiCo_2_O_4_ supported on three-dimensional hierarchical porous graphene-like material for highly effective oxygen evolution reaction

**DOI:** 10.1038/srep23398

**Published:** 2016-03-21

**Authors:** Wei-Yan Xia, Nan Li, Qing-Yu Li, Kai-Hang Ye, Chang-Wei Xu

**Affiliations:** 1 Guangzhou Key Laboratory for Environmentally Functional Materials and Technology, School of Chemistry and Chemical Engineering, Guangzhou University, Guangzhou 51006, China; 2 Guangxi Key Laboratory of Low Carbon Energy Materials, School of Chemistry and Pharmaceutical Sciences, Guangxi Normal University, Guilin 541004, China; 3 Department of Chemistry, Jinan University, Guangzhou 510632, China

## Abstract

A three-dimensional hierarchical porous graphene-like (3D HPG) material was synthesized by a one-step ion-exchange/activation combination method using a cheap metal ion exchanged resin as carbon precursor. The 3D HPG material as support for Au-NiCo_2_O_4_ gives good activity and stability for oxygen evolution reaction (OER). The 3D HPG material is induced into NiCo_2_O_4_ as conductive support to increase the specific area and improve the poor conductivity of NiCo_2_O_4_. The activity of and stability of NiCo_2_O_4_ significantly are enhanced by a small amount of Au for OER. Au is a highly electronegative metal and acts as an electron adsorbate, which is believed to facilitate to generate and stabilize Co^4+^ and Ni^3+^ cations as the active centres for the OER.

Electrochemical hydrogen evolution from water splitting by coupling renewable energy devices such as wind energy and solar energy with water electrolysis has attracted more and more attention in alkaline media due to continuous consumption of fossil fuels and ever-increasing environmental problems^1^. Hydrogen can be used as a fuel to get a reliable power for almost every application that fossil fuels are used. The hydrogen produced by electrolysis can be used for methanation of CO_2_, combustion processes, and conversion back into electricity by fuel cells^2^. In alkaline media, electrochemical water electrolysis consists of two half-reactions: the cathodic hydrogen evolution reaction (HER, 2H_2_O + 2e = 2OH^−^ + H_2_) and the anodic oxygen evolution reaction (OER, 4OH^−^ = 2H_2_O + 4e + O_2_). Of two half-reactions, the OER requires to form two oxygen-oxygen bonds in the four-electron redox processes by transfer protons and electrons, which results in more kinetically demand for the OER^3^,^4^. So, the OER needs relatively high overpotential at the anode, which is a major cause of high energy consumption. Thus, a lot of efforts have been devoted to explore the electrocatalysts with low OER overpotential.

The rutiletype oxides of RuO_2_ and IrO_2_ show the lowest OER overpotential, however theses oxides suffer from poor chemical stability in alkaline media and the high price and limited supply of Ru and Ir^5^,^6^. So other metal oxides such as Cu oxide^7^, Mn oxide^8^ have been developed. Among of all the oxide catalysts, particular attention has been paid to the cobalt oxide^9^,^10^ and nickel oxide^11^,^12^, due to their high abundance, low cost, small overpotential and fast kinetics of the OER. Many researchers have studied the other oxides to enhance the performance of OER for Ni oxide^13^,^14^ and Co oxide^15^,^16^. Trotochaud and his cooperators have reported that the conductivity of Ni oxide shows a > 30-fold increase with Fe oxide addition^13^. On the other hand, the presence of Fe alters the redox properties of Ni, causing a positive shift at the potential of Ni(OH)_2_/NiOOH redox reaction, a decrease in the average oxidation state of the Ni sites, and a concurrent increase in the activity of Ni cations for the OER^14^. The electrocatalytic synergism of mixed oxides of Co and Ni has been studied by many researchers^17^,^18^. Binary NiO_x_/CoO_x_-modified electrodes show high catalytic activity and marked stability which far exceed that obtained at the individual oxide-modified electrodes.

The nanohybrid materials have been used as efficient electrocatalysts for OER such as CoFe_2_O_4_^19^, CoMn_2_O_4_^20^, Ca_2_Mn_3_O_8_^21^, Co_2_MnO_4_^20^,^22^, Ca_2_Mn_2_O_5_^23^, CuCo_2_O_4_^24^, ZnCo_2_O_4_^25^ and CoMoO_4_^26^. The OER performance of NiCo_2_O_4_ spinel oxide has been studied in alkaline media and NiCo_2_O_4_ has high activity for OER^27^,^28^,^29^. We have reported that the activity of NiCo_2_O_4_ is much higher than that of NiO and Co_3_O_4_ for OER in 0.1 mol L^−1^ KOH^30^. Conductivity is an importance index for designing and developing available electrocatalysts for OER. With metallic conducting property of a conductivity of 10^−4^ S cm^−1^, RuO_2_ and IrO_2_ give the best OER activity^31^. However, many oxides such as NiCo_2_O_4_ suffer from low electrical conductivity. So, how to improve their poor intrinsic conductivity is still challenging for oxides. Therefore, carbon materials such as carbon nanotubes have been induced into oxides to improve the electrical conductivity^32^,^33^,^34^. Currently, graphene-based carbon materials including monolayer and multilayers nanosheets are highly promising materials as the new-generation supporting materials for electrocatalysts, owing to their high specific surface area, high electrical conductivity, and outstanding chemical and electrochemical stability^35^. The graphene as support for oxides such as MnO_x_^36^, CoO_x_^16^,^37^, CuFe oxide^38^, FeNi oxide^39^, NiCo oxide^40^, CoFe_2_O_4_^19^ and CuCo_2_O_4_^24^ has been reported for OER. Long and his cooperators reported that a synergy between the catalytic activity of the FeNi oxide and the enhanced electron transport arising from the graphene results in superior electrocatalytic properties for the OER^39^. The graphene supported NiCo_2_O_4_ has been reported for OER^41^,^42^,^43^. Zhao and coworkers have prepared an active catalyst composed of porous graphene and cobalt oxide (PGE–CoO), which has demonstrated high porosity, large specific surface area and fast charge transport kinetics^37^. The catalyst also exhibits excellent electrochemical performance towards OER with a low onset potential and high catalytic current density. The enhanced catalytic activity could be ascribed to porous structure, high electroactive surface area and strong chemical coupling between graphene and CoO nanoparticles. Moreover, this OER catalyst also shows good stability in the alkaline solution. The high performance and strong durability suggest that the porous structured composite is favorable and promising for water splitting. However, the intrinsic hydrophobic properties of graphitized basal plane structures cause a great difficulty in uniformly loading metal nanoparticles on the surface of graphene. Though the hydrophilicity of reduced graphene oxide (RGO) could be improved via introducing oxygen functional groups, their electronic conductivity is still insufficient due to their partly restored graphitic structures. Based on this fact, it is fundamental interest to develop the novel graphene-based carbon materials with high specific surface area, high electronic conductivity as well as strong affinity to foreign constituents, beyond the continuous development of hybrid architectures for electronics and various electrochemical systems^35^. Shen and coworkers have developed a novel active three-dimensional hierarchical porous graphene-like (3D HPG) material with hierarchical pores synthesized through an efficient ion-exchange-assisted synthesis route^44^. The 3D HPG material shows high electronic conductivity and strong cohesive force and distribution effects toward the catalyst nanoparticles^45^. The 3D HPG material can provide a highly conductive structure in conjunction with a large surface area to contact the MnO_2_ nanoparticles and effectively enhance the mechanical strength of the composite during volume changes as well as suppress the aggregation of MnO_2_ nanoparticles during Li-ion insertion/extraction^46^.

In recent years, a lot of efforts have been made to enhance the electrocatalytic activity catalysts and several strategies have been proposed. Among them, bifunctional mechanism, modified with highly electronegative metals, such as Pt^47^, Pd^48^ and Ru^49^, has been demonstrated as one of the most effective methods to improve the electrocatalytic efficiency. However, the high price and limited supply of Pt, Pd and Ru are major barriers to the development of OER catalysts using Pt-based, Pd-based and Ru-based catalysts. Scientists have pained more attention to Au because it is much more abundant and more available than Pt, Pd and Ru on the earth. Gold has been used to enhance the oxide activity of OER such as Co oxide^50^,^51^,^52^,^53^, Mn oxide^54^,^55^. A small amount of Au nanoparticles (<5%) in α-MnO_2_/Au catalysts significantly improves the catalytic activity up to 6 times compared with the activity of pure α-MnO_2_ for OER^54^. Bell and coworkers have developed noble metal-supported cobalt oxide and found that the OER activity of cobalt oxide deposited on Au is nearly three times higher than that of bulk Ir^51^. The Au/NiCo_2_O_4_ nanoarrays exhibit excellent OER activity, which is almost four times higher than that of Ir/C^56^.

In this paper, we focused on the development of high performance Au/NiCo_2_O_4_ catalysts supported on the 3D HPG material for the OER. It is widely accepted that 3D HPG is an outstanding matrix as support material with high electrical conductivity, good electrochemical stability, controllable specific surface areas as well as pore structure^46^,^57^.

## Results

**Figure 1:**
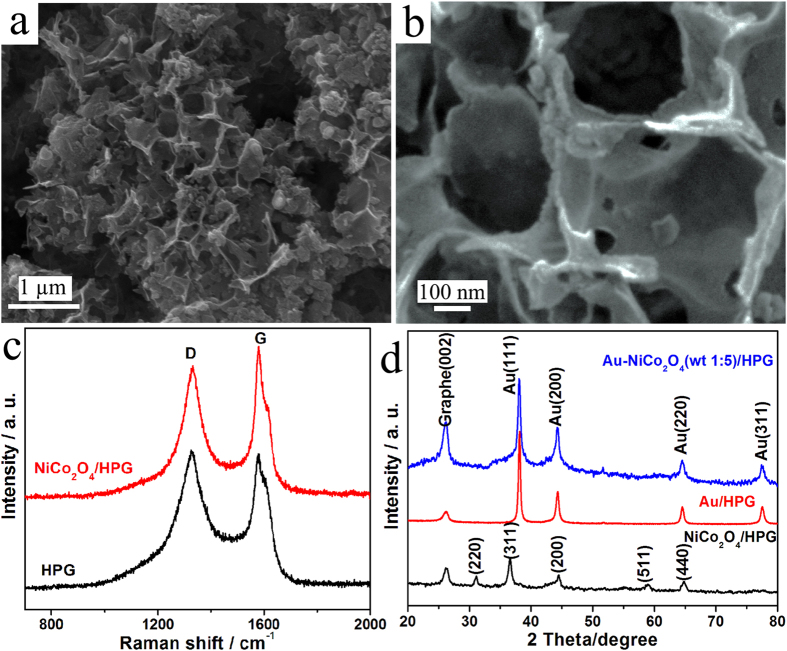
(**a,b**) SEM image for 3D HPG. (**c**) Raman spectra for HPG and NiCo_2_O_4_/HPG. (**d**) XRD patterns for NiCo_2_O_4_/HPG, Au/HPG and Au-NiCo_2_O_4_(wt 1:5)/HPG.

The morphology of the 3D HPG was characterized by scanning electron microscopy (SEM) as shown in Fig. 1a,b. The Fig. 1a shows a 3D interconnected porous structure with well-developed open macropores. The magnified SEM (Fig. 1b) exhibits sub-micrometer-sized pores. The thickness of the carbon sheet is about 6 nm. The degree of crystallinity of the HPG was characterized by Raman spectrum as shown in Fig. 1c. The G band peaked at 1578 cm^−1^ is related to the in-plane bond-stretching motion of the pairs of carbon sp^2^ atoms, which indicates the presence of crystalline graphene layers. The D band peak at 1329 cm^−1^ is assigned to disordered carbon and highly sensitive to graphitic defects within the graphite layers^58^. When the ratio of the peak intensity of D band to that of G band (*I*_D_/*I*_G_) is smaller, the degree of crystallinity will be higher^59^. Here the value of *I*_D_/*I*_G_ is 1.05 for HPG. The value of *I*_D_/*I*_G_ is 0.87 for NiCo_2_O_4_/HPG. The value of *I*_D_/*I*_G_ for NiCo_2_O_4_/HPG is a little lower than that of HPG. It may be attributable to the doping with NiCo_2_O_4_, which can induce defect sites and destruction in the carbon lattice, and lead to an increase in the degree of distortion^60^. X-ray diffraction (XRD) patterns for the Au/HPG, NiCo_2_O_4_/HPG, Au-NiCo_2_O_4_(wt 1:5)/HPG are shown in Fig. 1d. Diffraction peak at around 26.4° observed in all the samples is assigned to (002) plane of graphene. Diffraction peaks around 31.4°, 36.7°, 44.3°, 59.1° and 64.6° are assigned to the (220), (311), (200), (511) and (440) facets of NiCo_2_O_4_. In the case of NiCo_2_O_4_/HPG and Au-NiCo_2_O_4_(wt 1:5)/HPG, XRD pattern peaks of NiCo_2_O_4_ are in good agreement with the standard card (JCPDS no. 20-0781). The strong diffraction peaks at the Bragg angles of 38.1°, 44.4°, 64.5° and 77.5° correspond to the (111), (200), (220) and (311) facets of the face-centered-cubic (fcc) crystallite Au.

**Figure 2:**
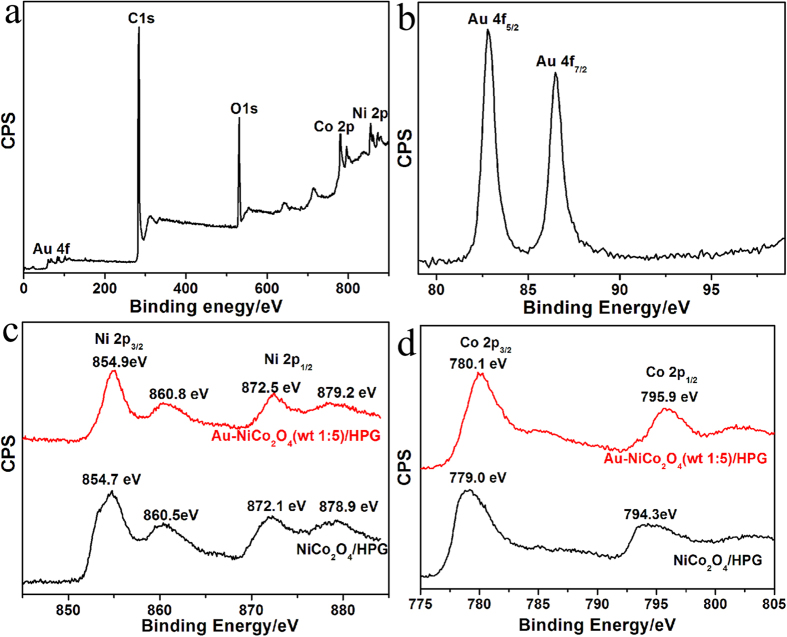
XPS spectra for Au-NiCo_2_O_4_(wt 1:5)/HPG. (**a**) survey, (**b**) Au 4f, (**c**) Ni 2p and (**d**) Co 2p.

Chemical bonding states in the Au-NiCo_2_O_4_(wt 1:5)/HPG were analyzed by X-ray photoelectron spectroscopy (XPS) as shown in Fig. 2. A survey spectrum of Au-NiCo_2_O_4_(wt 1:5)/HPG is shown in Fig. 2a and the peaks are corresponding to existence of Au 4f, C 1s, O 1s, Co 2p and Ni 2p. The binding energy values of XPS spectrum of Au 4f are 82.8 eV and 86.5 eV, which can be assigned to the Au 4f_5/2_ and Au 4f_7/2_ as shown in Fig. 2b, which are typical binding energy values of metallic Au^0^ species^61^. These data show that Au species attached to the surface of 3D HPG exist in the form of Au^0^. The binding energy of C 1s peak locates at 284.1 eV which is related to the graphitic carbon in 3D HPG as shown in Fig. 2a. The binding energy values of XPS spectrum of Ni 2p are 854.9 eV and 872.5 eV for Au-NiCo_2_O_4_(wt 1:5)/HPG as shown in Fig. 2c can be assigned to the Ni2p_3/2_ and Ni2p_1/2_, which can be assigned to Ni^2+^. The binding energy values of XPS spectrum of Co 2p are 780.1 eV and 795.9 eV for Au-NiCo_2_O_4_(wt 1:5)/HPG as shown in Fig. 2d can be assigned to the Co 2p_3/2_ and Co2p_1/2_, which can be assigned to Co^3+^. The relatively narrow peak width, the 2p_3/2_ to 2p_1/2_ separation of 15.9 eV, and the absence of any shake-up peak all reveal that no Co^2+^ exists in the NiCo_2_O_4_ phase^56^. It means that the Co cation is composed of lots of Co^3+^.

**Figure 3:**
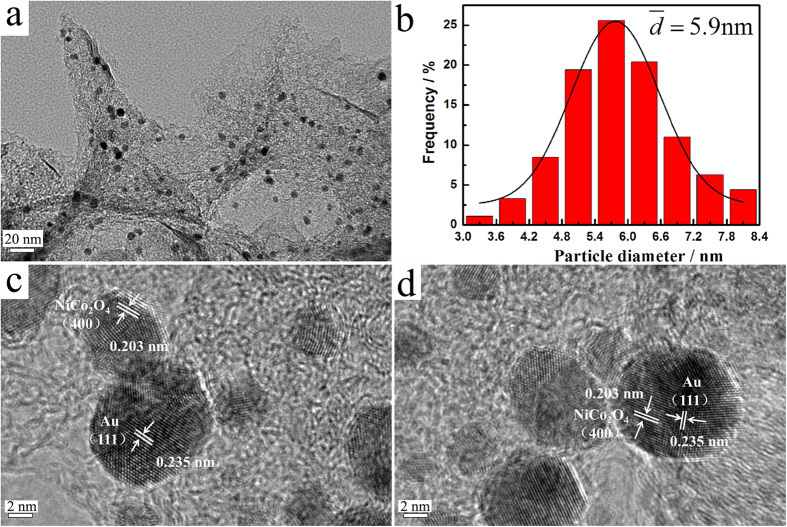
(**a**) TEM image, (**b**) particle size distribution histogram, (**c**) and (**d**) HRTEM images for Au-NiCo_2_O_4_(wt 1:5)/HPG.

**Figure 4:**
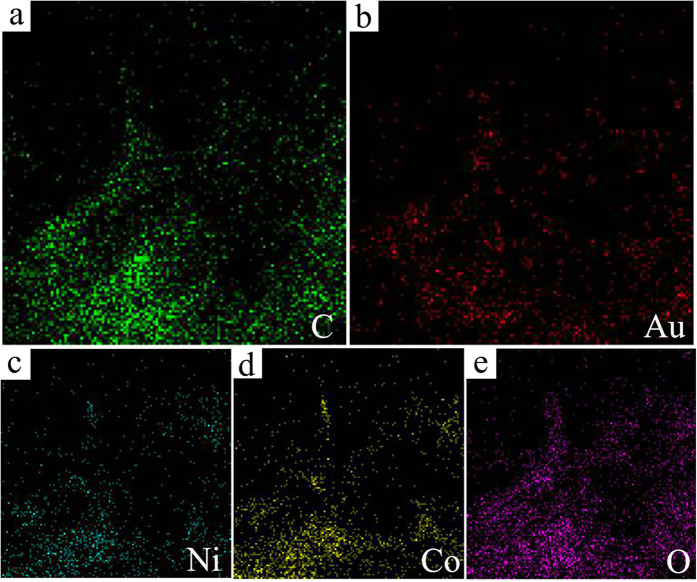


The typical high-resolution transmission electron microscopy (HRTEM) images of the Au-NiCo_2_O_4_(wt 1:5)/HPG are shown in Fig. 3. It can be observed that the catalyst particles are well dispersed on the surface of HPG with a narrow size distribution of 3–8 nm (Fig. 3a) and the average particle size is around 5.9 nm (Fig. 3b). The parallel fringe with a spacing of 0.203 nm is corresponding to the (400) plane of NiCo_2_O_4_ and the parallel fringe with a spacing of 0.235 nm is corresponding to the (111) plane of Au as shown in Fig. 3c,d. The nanoparticles of NiCo_2_O_4_ and Au contact each other or exit in one particle. The element mapping images (Fig. 4) show homogeneous C (Fig. 4a), Au (Fig. 4b), Ni (Fig. 4c), Co (Fig. 4d) and O (Fig. 4e) distributions in the Au-NiCo_2_O_4_(wt 1:5)/HPG (Fig. 3a) . The element mapping images also shows that the catalyst particles are well dispersed on the surface of HPG.

**Figure 5:**
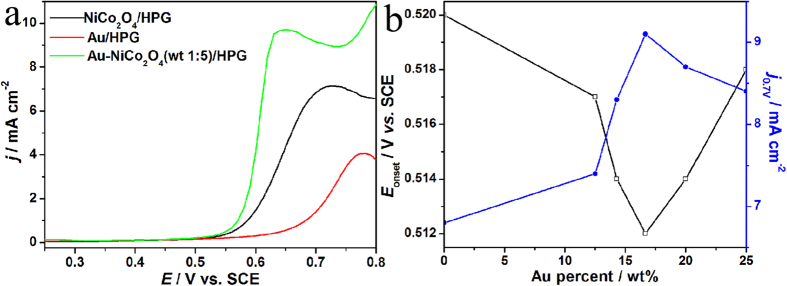
(**a**) LSV curves on NiCo_2_O_4_/HPG, Au/HPG and Au-NiCo_2_O_4_(wt 1:5)/HPG electrodes in 0.1 mol L^−1^ KOH with a sweep rate of 0.001 V s^−1^. (**b**) Plots of *E*_onset_ and *j*_0.7V_ in LSV curves as a function of the Au weight percent in Au-NiCo_2_O_4_/HPG with a total loading of 0.1 mg cm^−2^ on the electrodes.

In order to illustrate the advantages of the Au-NiCo_2_O_4_(wt 1:5)/HPG electrocatalyst, the OER activity of Au/HPG, NiCo_2_O_4_/HPG and Au-NiCo_2_O_4_(wt 1:5)/HPG is evaluated through linear sweep voltammetry (LSV) curves in 0.1 mol L^−1^ KOH with a sweep rate of 0.001 V s^−1^ as shown in Fig. 5a. The sum of loading for Au and NiCo_2_O_4_ on the electrodes was accurately controlled at 0.1 mg cm^−2^. The onset potential (*E*_onset_) of OER is 0.589 V on the Au/HPG electrode, 0.520 V on the NiCo_2_O_4_/HPG electrode, and 0.512 V on the Au-NiCo_2_O_4_(wt 1:5)/HPG electrode. The value of *E*_onset_ on the Au-NiCo_2_O_4_(wt 1:5)/HPG electrode is 8 and 77 mV lower than that on the NiCo_2_O_4_/HPG and Au/HPG electrodes. When the value of *E*_onset_ is lower, the OER happens easier. OER has the lowest value of *E*_onset_ on the Au-NiCo_2_O_4_(wt 1:5)/HPG electrode, so OER happens the most easily on the Au-NiCo_2_O_4_(wt 1:5)/HPG electrode. The values of current density at 0.7 V (*j*_0.7V_) are 1.4, 6.8 and 9.1 mA cm^−2^ on the Au/HPG, NiCo_2_O_4_/HPG and Au-NiCo_2_O_4_(wt 1:5)/HPG electrodes. The Au is poorly active for OER and the NiCo_2_O_4_ as a major electrocatalyst shows a considerable activity. The current density on the NiCo_2_O_4_/HPG improves remarkably by adding a small amount of Au. There is a synergistic effect between Au and NiCo_2_O_4_ because the value of *E*_onset_ is lower and the current density is higher on the Au-NiCo_2_O_4_(wt 1:5)/HPG electrode than that on the Au/HPG and NiCo_2_O_4_/HPG electrodes. The values of *E*_onset_ and *j*_0.7V_ of LSV on the Au-NiCo_2_O_4_/HPG catalysts with different weight percent of Au are compared as shown in Fig. 5b. The sum of loading for Au and NiCo_2_O_4_ on the electrodes was accurately controlled at 0.1 mg cm^−2^ in the Au-NiCo_2_O_4_/HPG electrode. The lowest value of *E*_onset_ is 0.512 V when weight ratio for Au to NiCo_2_O_4_ is 1: 5. The highest value of *j*_0.7V_ is 9.1 mA cm^−2^ when weight ratio for Au to NiCo_2_O_4_ is 1: 5. It can be seen that Au-NiCo_2_O_4_(wt 1:5)/HPG shows the highest activity for OER.

**Figure 6:**
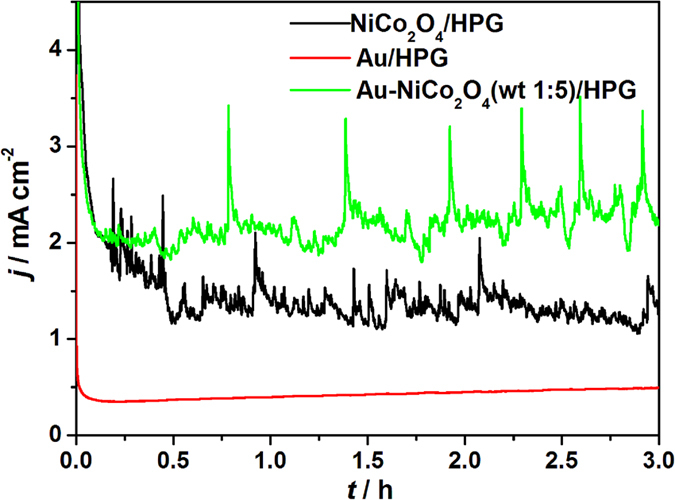


The stability of OER on the all electrodes is investigated by chronoamperometry. The chronoamperometry curves for OER in 0.1 mol L^−1^ KOH solution under a potential of 0.7 V was shown in Fig. 6. The current has a wave because the oxygen evolution on the NiCo_2_O_4_/HPG and Au-NiCo_2_O_4_(wt 1:5)/HPG electrodes, which also exhibits that NiCo_2_O_4_/HPG and Au-NiCo_2_O_4_(wt 1:5)/HPG catalysts have good activity for OER. When the OER happens, the oxygen bubbles will form on the surface of electrode and block the active sites on the surface of electrode, then the current of water oxidation will decrease. As the reaction is proceeding, oxygen bubbles grow up gradually, and the current of water oxidation also decreases. When the oxygen bubbles grow up enough to separate from the surface of electrode, the current of water oxidation will increase significantly due to the active sites on the surface of electrode are released for further electrochemical reaction. Until the end of the experiment, the oxidation current density on the Au-NiCo_2_O_4_(wt 1:5)/HPG electrode is 2.2 mA cm^−2^, which is 1.7 times as bigger as that on the NiCo_2_O_4_/HPG electrode (1.3 mA cm^−2^). The result shows that OER on the Au-NiCo_2_O_4_(wt 1:5)/HPG electrode has a higher current density than that on the NiCo_2_O_4_/HPG electrode with the same potential.

For further understanding of the intrinsic reaction of the OER performance on the Au-NiCo_2_O_4_(wt 1:5)/HPG catalyst, the XPS data of pure NiCo_2_O_4_/HPG and Au-NiCo_2_O_4_(wt 1:5)/HPG catalysts were compared as shown in Fig. 2c,d. The XPS data show that the binding energy value of Ni 2p has a 0.2~0.4 eV positively shift and that of Co 2p has a 1.1~1.6 eV positively shift after loading with Au. Au is a highly electronegative metal and acts as an electron adsorbate, which generates and stabilizes cobalt and nickel ions at higher oxidation states (e.g. Co^4+^ and Ni^3+^). Such a big shift in binding energy will promote the formation of Co^4+^ and Ni^3+^ cations. A general understanding is that the Co^4+^ and Ni^3+^ cations are the active centres for the OER. The presence of strong electrophilic Co^4+^ and Ni^3+^ cations will accelerate to form the OOH via nucleophilic reaction with O^62^. Depend on electrochemical oxidation, progressive oxidation from Co^3+^ to Co^4+^ and Ni^2+^ to Ni^3+^ is supposed as rate-limiting step, so the increased population of Co^4+^ and Ni^3+^ cations results in enhanced OER performance. Similar results were reported in OER using metal oxides (Co and Ni) with noble metals, where the noble metals generate and stabilize metal ions at higher oxidation states (e.g. Co^4+^ and Ni^3+^). Casella and his cooperators^63^ and Yeo and his cooperators^64^ demonstrated that the growth of Ni hydroxide on a gold electrode favors the oxide states of Ni^3+^ over Ni^2+^. Yeo and his cooperators also noted that the cobalt oxide deposited on Au electrodes exhibits a high occurrence of Co^4+^ species on the surface^52^. The enhanced activity was correlated to the electronegativity of noble metals.

In conclusion, a 3D HPG material was synthesized by a one-step ion-exchange/activation combination method using a cheap metal ion exchanged resin as carbon precursor. The 3D HPG material as support for Au-NiCo_2_O_4_ gives good activity and stability for OER. The 3D HPG material is induced into NiCo_2_O_4_ as conductive support to increase the specific area and improve the poor conductivity of NiCo_2_O_4_. The activity of and stability of NiCo_2_O_4_ significantly are enhanced by a small amount of Au for OER. The Au-NiCo_2_O_4_(wt 1:5)/HPG shows the highest activity for OER. The value of *E*_onset_ on the Au-NiCo_2_O_4_(wt 1:5)/HPG electrode is 8 and 77 mV lower than that on the NiCo_2_O_4_/HPG and Au/HPG electrodes. The values of *j*_0.7V_ are 1.4, 6.8 and 9.1 mA cm^−2^ on the Au/HPG, NiCo_2_O_4_/HPG and Au-NiCo_2_O_4_(wt 1:5)/HPG electrodes. Benefiting from the synergistic effect, the as-prepared Au-NiCo_2_O_4_/HPG catalyst shows significantly higher activity and better stability than NiCo_2_O_4_/HPG catalyst. The XPS data show that the binding energy value of Ni 2p has a 0.2~0.4 eV positively shift and that of Co 2p has a 1.1~1.6 eV positively shift after loading with Au. Au is a highly electronegative metal and acts as an electron adsorbate, which is believed to facilitate to generate and stabilize Co^4+^ and Ni^3+^ cations as the active centres for the OER.

## Methods

### Materials synthesis

The 3D HPG was synthesized by a one-step ion-exchange/activation combination method using a cheap metal ion exchanged resin as carbon precursor according to the Li method^44^. Firstly, the pretreated macroporous acrylic type cation exchange resin was impregnated with targeting ions of nickles in 0.05 mol L^−1^ nickel acetate solution for 6 h. Secondly, the exchange resin was washed with deionized water and dried at 333 K for 12 h. And then the exchanged resin was added into a 400 mL KOH/ethanol solution which contained 40 g KOH and stirred at 353 K for 6 h. After that, the mixture solution was dried at 343 K for 48 h and smashed by a disintegrator. Finally, the mixture was heated at 1123 K for 2 h in N_2_ atmosphere. When the resulted sample cooled down to room temperature, 3 mol L^−1^ HCl solution was added in it with a specific volume for more than 12 h with magnetic stirring. After that, the sample was repeated washed until the pH value was 7 and dried at 343 K. NiCo_2_O_4_/HPG was prepared through a typical heterogeneous reaction method. 1 mmol of Ni(NO_3_)_2_·6H_2_O, 2 mmol of Co(NO_3_)_2_·6H_2_O and 0.4811 g HPG were added into H_2_O (40 mL), followed by the addition of 5 mmol NH_4_F and 12 mmol urea. After being stirred for 1 h, the obtained mixture was transferred to a Teflon-lined stainless steel autoclave and heated to 393 K for 6 h. The resultant precipitate was washed several times with deionized water until the pH of the filtrate became 7 before being dried in a vacuum oven for 12 h. Finally the obtained powder was then annealed at 673 K for 2 h in air. The Au-NiCo_2_O_4_/HPG electrocatalysts were prepared by reduction of HAuCl_4_ solution on the NiCo_2_O_4_/HPG powders using an excess 0.01 mol L^−1^ NaBH_4_ solution.

### Electrode preparation

The electrocatalyst powders were dispersed in deionized water with 5 wt% PTFE (polytetrafluoroethylene) on the surface of a graphite rod with a geometric area of 0.33 cm^2^. The loading of carbon black and PTFE on the electrodes was accurately controlled at 0.23 mg cm^−2^ and 0.1 mg cm^−2^. The total loading of amount of Au and NiCo_2_O_4_ in the catalysts on the electrodes was accurately controlled at 0.1 mg cm^−2^.

### Characterization

XRD was carried out using a Panalytical X’Pert powder X-ray diffractometer with Cu Kα radiation (*λ* = 0.15418 nm). SEM images were obtained using a Quanta 400 FEG microscope (FEI Company). Transmission electron microscopy (TEM) images were carried out on a JEOL JEM-2010 (JEOL Ltd.). Raman spectroscopic measurements were carried out on a Raman spectrometer (Renishaw Corp., UK) using a He–Ne laser with a wavelength of 514.5 nm. XPS measurements were performed in an ESCALAB 250 spectrometer under vacuum (about 2 × 10^−9^ mbar). All electrochemical measurements were carried out in 0.1 mol L^−1^ KOH solution by using the solartron 1287 electrochemical work station using a standard three-electrode cell at 298 K. Solutions were freshly prepared before each experiment. A platinum foil (3.0 cm^2^) was used as counter electrode. All the potentials were measured versus a saturated calomel electrode (SCE, 0.241 V versus SHE) electrode. A salt bridge was used between the cell and the reference electrode.

## Additional Information

**How to cite this article**: Xia, W.-Y. *et al*. Au-NiCo_2_O_4_ supported on three-dimensional hierarchical porous graphene-like material for highly effective oxygen evolution reaction. *Sci. Rep.* **6**, 23398; doi: 10.1038/srep23398 (2016).

## References

[b1] Wang M. Y., Wang Z., Gong X. Z. & Guo Z. C. The intensification technologies to water electrolysis for hydrogen production–A review. Renew. Sust. Energ. Rev. 29, 573–588 (2014).

[b2] Schalenbach M. & Stolten D. High-pressure water electrolysis: Electrochemical mitigation of product gas crossover. Electrochim Acta 156, 321–327 (2015).

[b3] Surendranath Y., Kanan M. W. & Nocera D. G. Mechanistic studies of the oxygen evolution reaction by a cobalt-phosphate catalyst at neutral pH. J. Am. Chem. Soc. 132, 16501–16509 (2010). 20977209 10.1021/ja106102b

[b4] Bediako D. K., Surendranath Y. & Nocera D. G. Mechanistic studies of the oxygen evolution reaction mediated by a nickel–borate thin film electrocatalyst. J. Am. Chem. Soc. 135, 3662–3674 (2013). 23360238 10.1021/ja3126432

[b5] Paoli E. A. . Oxygen evolution on well-characterized massselected Ru and RuO_2_ nanoparticles. Chem. Sci. 6, 190–196 (2015). 10.1039/c4sc02685c PMC5424673 28553467

[b6] Casalongue H. G. S. . *In situ* observation of surface species on iridium oxide nanoparticles during the oxygen evolution reaction. Angew. Chem. Int. Ed. 53, 7169–7172 (2014). 10.1002/anie.201402311 24889896

[b7] Cheng N. Y. . Cu/(Cu(OH)_2_-CuO) core/shell nanorods array: *in-situ* growth and application as an efficient 3D oxygen evolution anode. Electrochim. Acta 163, 102–106 (2015).

[b8] Jin K. . Partially oxidized sub-10 nm MnO nanocrystals with high activity for water oxidation catalysis. Sci. Rep. 5, 10279; doi: 10.1038/srep10279 (2015). 25998696 PMC4441116

[b9] Liu Y. C., Koza J. A. & Switzer J. A. Conversion of electrodeposited Co(OH)_2_ to CoOOH and Co_3_O_4_, andcomparison of their catalytic activity for the oxygen evolution reaction. Electrochim. Acta 140, 359–365 (2014).

[b10] Zhao J. . Self-template construction of hollow Co_3_O_4_ microspheres from porous ultrathin nanosheets and efficient noble metal-free water oxidation catalysts. Nanoscale 6, 7255–7262 (2014). 24700250 10.1039/c4nr00002a

[b11] Stern L. A. & Hu X. L. Enhanced oxygen evolution activity by NiO_*x*_ and Ni(OH)_2_ nanoparticles. Faraday Discuss. 176, 363–379 (2014). 25406631 10.1039/c4fd00120f

[b12] Andersen N. I., Serov A. & Atanassov P. Metal oxides/CNT nano-composite catalysts for oxygenreduction/oxygen evolution in alkaline media. Appl. Catal. B–Environ. 163, 623–627(2015).

[b13] Trotochaud L., Young S. L., Ranney J. K. & Boettcher S. W. Nickel−iron oxyhydroxide oxygen-evolution electrocatalysts: The role of intentional and incidental iron incorporation. J. Am. Chem. Soc. 136, 6744–6753 (2014). 24779732 10.1021/ja502379c

[b14] Louie M. W. & Bell A. T. An investigation of thin-film Ni–Fe oxide catalysts for the electrochemical evolution of oxygen. J. Am. Chem. Soc. 135, 12329–12337 (2013). 23859025 10.1021/ja405351s

[b15] Zhang Y. . Hierarchical cobalt-based hydroxide microspheres for water oxidation. Nanoscale 6, 3376–3383 (2014). 24525520 10.1039/c3nr05193e

[b16] Zhao Y. F. . Graphene-Co_3_O_4_ nanocomposite as electrocatalyst with high performance for oxygen evolution reaction. Sci. Rep. 5, 7629; doi: 10.1038/srep07629 (2015). 25559459 PMC4284504

[b17] Liang H. F. . Hydrothermal continuous flow synthesis and exfoliation of NiCo layered double hydroxide nanosheets for enhanced oxygen evolution catalysis. Nano Lett. 15, 1421–1427 (2015). 25633476 10.1021/nl504872s

[b18] Zhu C. Z. . Nickel cobalt oxide hollow nanosponges as advanced electrocatalysts for the oxygen evolution reaction. Chem. Commun. 51, 7851–7854 (2015). 10.1039/c5cc01558h 25855058

[b19] Bian W. Y., Yang Z. R., Strasser P. & Yang R. Z. A CoFe_2_O_4_/graphene nanohybrid as an efficient bi-functional electrocatalyst for oxygen reduction and oxygen evolution. J. Power Sources 250, 196–203 (2014).

[b20] Menezes P. W. . Cobalt–manganese-based spinels as multifunctional materials that unify catalytic water oxidation and oxygen reduction reactions. ChemSusChem 8, 164–171 (2015). 25394186 10.1002/cssc.201402699

[b21] Ramíez A., Bogdanoff P., Friedrich D. & Fiechter S. Synthesis of Ca_2_Mn_3_O_8_ films and their electrochemical studies for the oxygen evolution reaction(OER) of water. Nano Energy 1, 282–289 (2012).

[b22] Wang D. D., Chen X., Evans D. G. & Yang W. S. Well-dispersed Co_3_O_4_/Co_2_MnO_4_ nanocomposites as a synergistic bifunctional catalyst for oxygen reduction and oxygen evolution reactions. Nanoscale 5, 5312–5315 (2013). 23681343 10.1039/c3nr00444a

[b23] Kim J., Yin X., Tsao K. C., Fang S. H. & Yang H. Ca_2_Mn_2_O_5_ as oxygen-deficient perovskite electrocatalyst for oygen evolution reaction. J. Am. Chem. Soc. 136, 14646–14649 (2014). 25295698 10.1021/ja506254g

[b24] Bikkarolla S. K. & Papakonstantinou P. CuCo_2_O_4_ nanoparticles on nitrogenated graphene as highly efficientoxygen evolution catalyst. J. Power Sources 281, 243–251 (2015).

[b25] Kim T. W., Woo M. A., Regis M. & Choi K. S. Electrochemical synthesis of spinel type ZnCo_2_O_4_ electrodes for use as oxygen evolution reaction catalysts. J. Phys. Chem. Lett. 5, 2370–2374 (2014). 26279561 10.1021/jz501077u

[b26] Yu M. Q., Jiang L. X. & Yang H. G. Ultrathin nanosheets constructed CoMoO_4_ porous flowers with high activity for electrocatalytic oxygen evolution. Chem. Commun. 51, 14361–14364 (2015). 10.1039/c5cc05511c 26269035

[b27] Wang J., Qiu T., Chen X., Lu Y. L. & Yang W. S. Hierarchical hollow urchin-like NiCo_2_O_4_ nanomaterial as electrocatalyst for oxygen evolution reaction in alkaline medium. J. Power Sources 268, 341–348 (2014).

[b28] Jin C., Lu F. L., Cao X. C., Yang Z. R. & Yang R. Z. Facile synthesis and excellent electrochemical properties of NiCo_2_O_4_ spinel nanowire arrays as a bifunctional catalyst for the oxygen reduction and evolution reaction. J. Mater. Chem. A 1, 12170–12177 (2013).

[b29] Chen R., Wang H. Y., Miao J. W., Yang H. B. & Liu B. A flexible high-performanceoxygenevolution electrode with three-dimensional NiCo_2_O_4_ core-shellnanowires. Nano Energy 11, 333–340 (2015).

[b30] Su Y. Z. . NiCo_2_O_4_/C prepared by One-step Intermittent Microwave Heating Method for Oxygen Evolution Reaction in Water Splitter. J. Alloys Compd. 617, 115–119 (2014).

[b31] Reier T., Oezaslan M. & Strasser P. Electrocatalytic oxygen evolution reaction (OER) on Ru, Ir, and Pt catalysts: A comparative study of nanoparticles and bulk materials. ACS Catal. 2, 1765–1772 (2012).

[b32] Cheng Y., Shen P. K. & Jiang S. P. NiOx nanoparticles supported on polyethylenimine functionalized CNTs as efficient electrocatalysts for supercapacitor and oxygen evolution reaction. Int. J. Hydrogen Energy 39, 20662–20670 (2014).

[b33] Li B. B. . MoO_2_–CoO coupled with a macroporous carbon hybrid electrocatalyst for highly efficient oxygen evolution. Nanoscale 7, 16704–16714 (2015). 26399728 10.1039/c5nr04666a

[b34] Tang H. L. . Enhancedsupercapacitiveperformance on TiO_2_@C coaxialnano-rodarraythrough a bio-inspiredapproach. Nano Energy 15, 75–82 (2015).

[b35] Li Z. S., Li Y. Y., Jiang S. P., He G. Q. & Shen P. K. Novel graphene-like nanosheet supported highly active electrocatalysts with ultralow Pt loadings for oxygen reduction reaction. J. Mater. Chem. A 2, 16898–16904 (2014).

[b36] Chen S., Duan J. J., Han W. & Qiao S. Z. A Graphene–MnO_2_ framework as a new generation of three-dimensional oxygen evolution promoter. Chem. Commun. 50, 207–209 (2014). 10.1039/c3cc47665k 24217532

[b37] Zhao Y. F. . Porous graphene wrapped CoO nanoparticles for highly efficient oxygen evolution. J. Mater. Chem. A 3, 5402–5408 (2015).

[b38] Geng J., Kuai L., Kan E. J., Wang Q. & Geng B. Y. Precious-metal-free Co–Fe–O/rGO synergetic electrocatalysts for oxygen evolution reaction by a facile hydrothermal route. ChemSusChem 8, 659–664 (2015). 25572639 10.1002/cssc.201403222

[b39] Long X. . A strongly coupled graphene and FeNi double hydroxide hybrid as an excellent electrocatalyst for the oxygen evolution reaction. Angew. Chem. Int. Ed. 53, 7584–7588 (2014). 10.1002/anie.201402822 24910179

[b40] Chen S., Duan J. J., Jaroniec M. & Qiao S. Z. Three-dimensional N-doped graphene hydrogel/NiCo double hydroxide electrocatalysts for highly efficient oxygen evolution. Angew. Chem. Int. Ed. 52, 13567–13570 (2013). 10.1002/anie.201306166 24346940

[b41] Chen S. & Qiao S. Z. Hierarchically porous nitrogen-doped graphene NiCo_2_O_4_ hybrid paper as an advanced electrocatalytic water-splitting material. Acs Nano 7, 10190–10196 (2013). 24090468 10.1021/nn404444r

[b42] Lee D. U., Kim B. J. & Chen Z. W. One-pot synthesis of a mesoporous NiCo_2_O_4_ nanoplatelet and graphene hybrid and its oxygen reduction and evolution activities as an efficient bi-functional electrocatalyst. J. Mater. Chem. A 1, 4754–4762 (2013).

[b43] Gao Z., Yang W. L., Wang J., Song N. N. & Li X. D. Flexible all-solid-state hierarchical NiCo_2_O_4_/porous graphene paper asymmetric supercapacitors with an exceptional combination of electrochemical properties. Nano Energy 13, 306–317 (2015).

[b44] Li Y. Y., Li Z. S. & Shen P. K. Simultaneous formation of ultrahigh surface area and three-dimensional hierarchical porous graphene-like networks for fast and highly stable supercapacitors. Adv. Mater. 25, 2474–2480 (2013). 23495046 10.1002/adma.201205332

[b45] Li Y. Y., Zhang H. Y. & Shen P. K. Ultrasmall metal oxide nanoparticles anchored on three-dimensional hierarchical porous gaphene-like networks as anode for high-performance lithium ion batteries. Nano Energy 13, 563–572 (2015).

[b46] Li Y. Y., Zhang Q. W., Zhu J. L., Wei X. L. & Shen P. K. An extremely stable MnO_2_ anode incorporated with 3D porous graphene-like networks for lithium-ion batteries. J. Mater. Chem. A 2, 3163–3168 (2014).

[b47] Han X. P. . Hydrogenated uniform Pt clusters supported on porous CaMnO_3_ as a bifunctional electrocatalyst for enhanced oxygen reduction and evolution. Adv. Mater. 26, 2047–2051 (2014). 24818256 10.1002/adma.201304867

[b48] Li Z. Y., Liu Z. L., Liang J. C., Xu C. W. & Lu X. H. Facile synthesis of Pd-Mn_3_O_4_/C as high-efficient electrocatalyst for oxygen evolution reaction. J. Mater. Chem. A 2, 18236–18240 (2014).

[b49] Berenguer R., Sieben J. M., Quijada C. & Morallón E. Pt- and Ru-doped SnO_2_−Sb anodes with high stability in alkaline medium. ACS Appl. Mater. Interfaces 6, 22778–22789 (2014). 25453898 10.1021/am506958k

[b50] Zhuang Z. B., Sheng W. C. & Yan Y. S. Synthesis of monodispere Au@Co_3_O_4_ core-shell nanocrystals and their enhanced catalytic activity for oxygen evolution reaction. Adv. Mater. 26, 3950–3955 (2014). 24687515 10.1002/adma.201400336

[b51] Walton A. S. . Interface controlled oxidation states in layered cobalt oxide nanoislands on gold. Acs Nano 9, 2445–2453 (2015). 25693621 10.1021/acsnano.5b00158

[b52] Yeo B. S. & Bell A. T. Enhanced activity of gold-supported cobalt oxide for the electrochemical evolution of oxygen. J. Am. Chem. Soc. 133, 5587–5593 (2011). 21413705 10.1021/ja200559j

[b53] Zhang Y., Cui B., Qin Z. T., Lin H. & Li J. B. Hierarchical wreath-like Au–Co(OH)_2_ microclusters for water oxidation at neutral pH. Nanoscale 5, 6826–6833 (2013). 23771130 10.1039/c3nr01735d

[b54] Kuo C. H. . Understanding the role of gold nanoparticles in enhancing the catalytic activity of manganese oxides in water oxidation reactions. Angew. Chem. Int. Ed. 54, 2345–2350 (2015). 10.1002/anie.201407783 25284796

[b55] Gorlin Y. . Understanding interactions between manganese oxide and gold. That lead to enhanced activity for electrocatalytic water oxidation. J. Am. Chem. Soc. 136, 4920–4926 (2014). 24661269 10.1021/ja407581w PMC4004245

[b56] Liu X. J., Liu J. F., Li Y. P., Li Y. J. & Sun X. P. Au/NiCo_2_O_4_ arrays with high activity for water oxidation. ChemCatChem 6, 2501–2506 (2014).

[b57] Gao M. R. . Nitrogen-doped graphene supported CoSe_2_ nanobelt composite catalyst for efficient water oxidation. Acs Nano 8, 3970–3978 (2014). 24649855 10.1021/nn500880v

[b58] Hassan S., Suzuki M., Mori S. & El-Moneim A. A. MnO_2_/carbon nanowalls composite electrode for supercapacitor application. J. Power Sources 249, 21–27 (2014).

[b59] Sadezky A., Muckenhuber H., Grothe H., Niessner R. & Pöschl U. Raman microspectroscopy of soot and related carbonaceous materials: Spectral analysis and structural information. Carbon 43, 1731–1742 (2005).

[b60] Zhu J. L., Jiang S. P., Wang R. H., Shi K. Y. & Shen P. K. One-pot synthesis of a nitrogen and phosphorusdual-doped carbon nanotube array as a highly effective electrocatalyst for the oxygen reduction reaction. J. Mater. Chem. A 2, 15448–15453 (2014).

[b61] Park E. D. & Lee J. S. Effects of pretreatment conditions on CO oxidation over supported Au catalysts. J. Catal. 186, 1–11 (1999).

[b62] Man I. C. . Universality in oxygen evolution electrocatalysis on oxide surfaces. ChemCatChem 3, 1159–1165 (2011).

[b63] Casella I. G., Guascito M. R. & Sannazzaro M. G. Voltammetric and XPS investigations of nickel hydroxide electrochemically dispersed on gold surface electrodes. J. Electroanal. Chem. 462, 202–210 (1999).

[b64] Yeo B. S. & Bell A. T. *In situ* Raman study of nickel oxide and gold-supported nickel oxide catalysts for the electrochemical evolution of oxygen. J. Phys. Chem. C 116, 8394–8400 (2012).

